# Perinatal outcomes in women referred to the West Virginia University Assist Connect and Encourage (ACE) – A program of the Drug Free Moms and Babies Project (DFMB) for women with substance use during pregnancy

**DOI:** 10.1016/j.pmedr.2023.102312

**Published:** 2023-07-04

**Authors:** Omar Dueñas-Garcia, Robinson Lindsey, Marshall Elly, Lemon Kelly, Breyel Janine, Umer Amna, Hercules Catherine, Lilly Christa

**Affiliations:** a West Virginia University, Morgantown, USA; b West Virginia Perinatal Partnership, Charleston, WV, USA

**Keywords:** Pregnancy, Substance abuse, Perinatal outcomes, Neonatal abstinence syndrome, Preterm labor, Controlled substances

## Abstract

The main objective of this study was to analyze perinatal outcomes of women with substance use disorder in pregnancy who participated in the West Virginia University Medicine Drug Free Moms and Babies Assist Connect and Encourage program (DFMB/ACE) compared to women who were not enrolled in the program. This was a retrospective cohort study conducted in an l Academic Tertiary Care Center. Women who enrolled in DFMB/ACE services from 2018 to 2019 were termed as the intervention group. Their outcomes were compared to 734 women who delivered at the hospital between July 2015 and December 2019 with a positive urine drug screen on admission but were not enrolled in the DFMB/ACE program.

Perinatal outcomes evaluated include low birth weight, preterm labor, neonatal intensive care unit (NICU) admission, preterm birth, neonatal abstinence syndrome (NAS), cesarean delivery, cord toxicology result, hepatitis c prevalence, and breastfeeding at the time of discharge. Participants of the DFMB/ACE group had a lower risk of having a preterm birth (OR = 0.56 95% CI 0.36–0.86) compared to the control group. The DFMB/ACE group had higher birth weight compared to the non-DFMB group (2865.7 g. vs. 2657.9 g, p = 0.004). NICU admission, NAS, mode of delivery, cord toxicology, and breastfeeding rates were not statistically different between the two groups. Hepatitis C rates were significantly higher in the intervention vs. non-intervention group (OR = 2.74 95 %CI 1.80 – 4.16). We concluded that the DFMB/ACE program interventions for women with substance use in pregnancy improves some perinatal outcomes for mothers and their newborns.

## Highlights

• Participants of the DFMB/ACE group had a lower risk of having a preterm birth.
• Participants of the DFMB/ACE group had a higher birth weight.
• Patients with private insurance had more tobacco use compared to the DFMB/ACE program.
• The DFMB/ACE provides good support to women with fewer resources.

## 1 Background

Appalachia, and specifically West Virginia (WV), has been disproportionately impacted by the opioid epidemic. As of 2020, 13% of West Virginians over the age of 26 meet the diagnostic criteria for substance use disorder (SUD); among those between the ages of 18 and 25 years, the prevalence is over 30% (Center). Women of childbearing age represent a particularly at-risk population for substance use. Data from the Substance Abuse and Mental Health Service Administration (SAMHSA) reports that in 2016, 13.2% of women of childbearing age (15 to 44) and 6.3% of pregnant women used illicit drugs (Lipari et al., 2013). Maternal opioids and polysubstance in pregnancy are associated with poor perinatal outcomes including preterm birth, low birth weight, severe Neonatal Abstinence Syndrome (NAS), and NICU admission (Helen et al., 2022). Prematurity and low birth weight can lead to long-term complications including infection, cardiovascular complications, and learning disabilities. (Pravia, 2020) NAS in infants often leads to increased irritability, breathing problems, difficulty feeding, and poor weight gain (Fingar et al., 2006). Studies have also shown that infants who are diagnosed with NAS after birth are at higher risk for long-term developmental and educational delays (Fill et al. 2018). Despite those findings, NAS should be considered a treatable condition that is not inherently harmful, especially when NAS results from prescribed medications. The long-term care for NAS infants may be costly to hospitals, families, and payers (Fingar et al., n.d.).

A study by Stitely et al., in 2009 evaluated umbilical cord samples from eight WV birthing facilities and found that nearly 20% of all newborns were exposed to illicit or controlled substances in utero (Stitely et al., 2010). In response to these findings, the WV Perinatal Partnership developed the Drug Free Moms and Babies (DFMB) project. (Lilly et al., 2019). The project seeks to improve perinatal outcomes by providing prevention and early intervention, addiction treatment, and recovery services. This study focuses on the program outcomes of one of the DFMB programs located in north central West Virginia: The Assist, Connect, and Encourage (ACE) program. For clarity, it will be referred to as DFMB/ACE within this article, while the broad statewide intervention will be referred to as DFMB. The DFMB/ACE program is located within the West Virginia University Medicine Obstetrics and Gynecology and provides services for a large level IV tertiary care center in Morgantown, West Virginia. This study examined whether participation in the DFMB/ACE program was associated with better perinatal outcomes.

## 2 Material and methods

### 2.1 Description of the intervention

The DFMB/ACE team aims to identify women with substance use disorder before or during the early stages of their pregnancy. At the initial prenatal visit for all women, a state-required West Virginia Prenatal Screening Instrument (PRSI) form is completed, which includes self-reported substance use-related questions (Appendix A). A thorough history is also obtained by the obstetrics (OB) provider as a routine component of care, which includes questions regarding maternal substance and medication use. Pregnant women with self-reported substance use on either screening modality are invited to enroll and participate in the DFMB/ACE program. Participation in the program is free and voluntary. Consent for review of chart data is obtained upon enrollment. Patients can be enrolled in the program at any point during the antepartum or postpartum periods. Women with a history of substance use who are currently in recovery/remission are also encouraged to participate. Routine urine drug screening occurs as clinically appropriate; participants do not experience increased costs or more frequent urine drug screening because of participation. Engagement in treatment programs, including those using medication for opioid use disorder when needed, is facilitated. Members of the DFMB/ACE team help to connect participants to treatment that meets their needs. Participants are encouraged to engage in recovery-based meetings, such as Celebrate Recovery or Narcotics Anonymous, and are referred to local and online meetings and support groups regularly. A program coordinator completes ongoing needs assessments and referrals to social services.

A key component of program success is access to a Peer Recovery Support Specialist via phone/text for immediate support needs. Additional benefits from the program include health education, referral to community resources, including WIC, home visitation programs, and other social services, referrals to substance use and mental health treatment services, and coordination to ensure these services are accessed. Patients are followed for up to 2 years after the delivery of the infant by the peer recovery support specialist and the number of follow-up visits range from monthly or less to 2–3 times per week, including in-person, texting, and phone sessions.

### 2.2 Data abstraction

This retrospective cohort study used data abstracted from electronic medical records for all mothers who delivered at West Virginia University Medicine – Ruby Memorial Hospital between July 2015 and December 2019. The West Virginia University Institutional Review Board approved this research project. For this study, the non-intervention group included all subjects who delivered at West Virginia University Medicine Hospital in Morgantown WV, between July 2015 to December 2019 (n = 6754), had positive urine toxicology at admission (n = 734, 10.86%), and did not enroll in the DFMB/ACE program.

### 2.3 Outcomes

Due to a change in the database, outcomes for participants in the DFMB/ACE program (intervention group) could not be evaluated for the same period as the non-intervention group. The intervention group includes those women with positive urine toxicology results at labor and delivery who were enrolled in the DFMB/ACE program from 2018 to 2019. For the intervention group, existing data collected by the DFMB program was used for this study. The DFMB/ACE project coordinator utilized the master list of program participants to cross-reference the database and ensure there were no duplicate entries within the two groups.

Designated site personnel entered de-identified DFMB participants’ information using the Research Electronic Data Capture (REDCap) database hosted by the West Virginia Clinical Translational Science Institute (WVCTSI) [9]. Data entered into REDCap included demographic characteristics, medical and substance use histories, services received, and maternal and neonatal outcomes. The WVU DFMB/ACE birth cohort (intervention group) from 2018 to 2019 was compared to non-ACE/DFMB participants (non-intervention group) who delivered at WVU Medicine Ruby Memorial between 2015 and 2019. Specific maternal and fetal outcomes included NICU admission, pre-term delivery, NAS diagnosis, C-section rates, cord tissue toxicology, and breastfeeding at discharge. Additional continuous outcomes include infant birth weight in grams and gestational age in weeks. Neonatal abstinence syndrome was diagnosed by the NICU team members based on maternal exposure to illicit substances, urine or cord toxicology, and clinical features.

Prenatal care (PNC) was categorized as adequate, intermediate, and inadequate according to the latest American College of Obstetricians and Gynecologists (ACOG) definitions of adequate, intermediate, and inadequate prenatal care (PNC) participation (“ACOG Committee Opinion. Scope of Services for Uncomplicated Obstetric Care. Number 175, September 1996 (Replaces No. 79, January 1990). American College of Obstetricians and Gynecologists,” 1996). Attendance/participation in PNC was deemed inadequate if it was initiated after the 4th month and if there were < 50% of the recommended visits. Care was delineated as intermediate if they attended 50–79% of all recommended visits and adequate if ≥ 80% of all recommended visits were attended. At least ten PNC visits are recommended for routine prenatal care (“ACOG Committee Opinion. Scope of Services for Uncomplicated Obstetric Care. Number 175, September 1996 (Replaces No. 79, January 1990). American College of Obstetricians and Gynecologists,” 1996).

### 2.4 Statistical analysis

All analyses were conducted using SAS 9.4. Descriptive statistics were conducted and included frequency and valid percentages for categorical data and means and standard deviations for continuous data. Median and range are presented for non-parametric continuous data. Bivariate group differences were tested using independent sample t-tests, Mann-Whitney two-sample Wilcoxon, odds ratio with 95% confidence limit, or Fisher’s exact test depending on the variable type. General linear models were conducted for continuous outcomes (e.g., gestational age, infant weight) and logistic regressions for binary outcomes to adjust for covariates. For the logistic regression model, we adjusted for insurance type, employment status, education, and marital status, and for the linear model we adjusted based on insurance type, employment status, education, and marital status. Alpha was set to 0.05 unless otherwise specified.

## 3 Results

The characteristics of the intervention group (DFMB/ACE, n = 134) and the non-intervention group (non-DFMB/ACE, n = 734) are detailed in Table 1. Both groups had a high percentage of inadequate PNC. More than 70% of women were between 25–––44 years old and had less than or equal to a high school education. As commonly reported in the Appalachia region, most of the women in both groups identified themselves as white non-Hispanic. A majority of the women were enrolled in Medicaid health insurance in the DFMB/ACE group (94%), with only 3% covered by private health insurance. Those in the non– DFMB/ACE group were more likely to have private insurance (28.2%).

**Table 1:** Population characteristics and comparison of non-DFMB (N = 734) and DFMB (N = 134) women.

**Population characteristics**	**WVU non-DFMB (n = 734)**		**WVU DFMB (n = 134)**		**Chi-square**
	**N**	**%**	**N**	**%**	**p-value**
Prenatal Care					0.06
Inadequate	356	50.4	49	39.8	
Intermediate	48	6.8	13	10.6	
Adequate	302	42.8	61	49.6	
Age					0.51
≤17	9	1.2	0	0	
18–24	209	28.6	35	26.1	
25–44	513	70.1	99	73.9	
45–64	1	0.1	0	0	
Insurance					<0.0001
Private	205	28.2	4	3	
Medicaid	508	70	124	93.9	
Self-pay/Other	13	1.8	4	3	
Education					<0.0001
Less than HS	101	21.4	30	23.6	
HS	223	47.2	56	44.1	
More than HS	149	31.4	41	32.3	
Employed					0.04
Unemployed	394	64.6	87	73.7	
Part-time	29	4.8	8	6.8	
Full-time	187	30.7	23	19.5	
Race					0.45
White	704	96.2	127	94.8	
Other	28	3.8	7	5.2	
Ethnicity					0.59
Hispanic	7	1	2	1.5	
Non-Hispanic	711	99	132	98.5	
Marital status					<0.0001
Married	221	30.9	9	6.7	
Unmarried	495	69.1	125	93.3	
Substance use*					
Alcohol	32	4.4	3	2.2	0.25
Tobacco	462	63.1	55	41	<0.0001
Cannabis	285	38.9	13	9.7	<0.0001
Stimulant	67	9.2	34	25.4	<0.0001
Opioids/narc	432	59	69	51.5	0.1
Depressants**	73	10	7	5.2	0.08
Other	116	15.9	3	2.2	<0.0001
Poly-substance use					<0.0001
Yes	489	66.8	59	44	
No	243	33.2	75	56	

* % are of sample and do not add to 100%.

** Benzodiazepines, barbiturates.

Abbreviations: HS-High School.

Polysubstance use includes more than one of any substance category specified.

Other includes any other type not otherwise specified (e.g., inhalants, steroids).

Most of the DFMB/ACE participants (intervention group) were unmarried (93.3%) compared to the non-intervention group (69.1%). Opioids and tobacco were the most used drugs during pregnancy for both groups (Table 1). Alcohol, tobacco, cannabis, opioids, depressants, and polysubstance use rates were lower in the intervention group compared to the non-intervention group. Overall, the non-DFMB/ACE group had a higher prevalence of polysubstance use. Stimulant rates were higher in the DFMB/ACE group compared to the non-intervention group (Table 1). In the DFMB/ACE group, 70.2% of the women were in a MOUD program compared to 35% in the non-DFMB/ACE group. Outcomes are displayed in Table 2, Table 3, Table 4. Participants in the DFMB/ACE program had fewer preterm deliveries with an odds ratio (OR) of 0.56 (95% CI 0.36–0.86). This difference was also consistent in extremely preterm (<28 weeks) and very preterm (28–32 weeks) deliveries (Table 4). The mean gestational age (37.26 vs. 36.42) and infant birth weight (2865.7 vs. 2657.9) were significantly higher in the DFMB/ACE group compared to the non-DFMB group (Table 3).

**Table 2:** Comparison of outcomes in the intervention group (DFMC/ACE) to the non-intervention group (WVU-non-DFMB).

Categorical Outcomes	WVU non-DFMB (n = 734)	WVU DFMB (n = 134)	Odds Ratio	95% CL lower	95% CL upper	p-value	Adj. OR	p-value
NICU								
Yes	348 (49.2%)	57 (44.9%)	0.84	0.58	1.23	0.37	0.81	0.41
No	359 (50.8%)	70 (55.1%)						
Preterm								
Yes	257 (35.0%)	31 (23.1%)	0.56	0.36	0.86	0.007	0.53	0.02
No	477 (65.0%)	103 (76.9%)						
NAS								
Yes	313 (44.5%)	53 (42.4%)	0.92	0.62	1.35	0.66	0.67	0.12
No	390 (55.5%)	72 (57.6%)						
Method delivery								
Vaginal	417 (58.8%)	83 (65.4%)	1.32	0.89	1.96	0.17	1.16	0.56
C-section	292 (41.2%)	44 (34.6%)						
Positive cord tissue								
Yes	173 (25.0%)	38 (31.2%)	1.36	0.89	2.07	0.15	0.84	0.53
No	519 (75.0%)	84 (68.9%)						
Breastfeed at discharge								
Yes	282 (40.1%)	57 (44.9%)	1.22	0.83	1.78	0.31	1.61	0.06
No	421 (59.9%)	70 (55.1%)						
MOUD								
Yes	257 (35.0%)	94 (70.2%)	4.36	2.92	6.51	<0.0001	5.18	<0.0001
No	477 (65.0%)	40 (29.9%)						
Hepatitis C								
Yes	128 (17.66%)	44 (36.97%)	2.74	1.80	4.16	<0.0001	1.99	0.0151
No	597 (82.34%)	75 (63.03%)						

Abbreviations: NICU-neonatal intensive care unit. NAS –neonatal abstinence syndrome. MOUD – medication for opioid use disorder.

Adjusted for insurance type, employment status, education, and marital status.

**Table 3:** Perinatal continuous outcomes by gestational age and infant birth weight.

	**WVU non-DFMB**	**WVU DFMB**	**t-value**	**DF**	**p-value**	**Adj. p-value**
Gestational age n	701	129				
mean (SD)	36.42 (3.63)	37.26 (3.32)	−2.45	828	0.015	0.0035
Infant weight (grams) n	705	127				
mean (SD)	2657.9 (759.0)	2865.7 (713.6)	−2.87	830	0.004	0.0008

Adjusted for insurance type, employment status, education, and marital status in a general linear model.

**Table 4:** Perinatal outcomes by gestational age and weight.

	**Extreme pre-term (<28 weeks)**	**Very pre-term (28**–**32 weeks)**	**Preterm (33**–**36 weeks)**	**Term (37 weeks + )**	**Fisher’s exact p-value**
WVU non-DFMB (n = 701)	26 (3.7%)	61 (8.7%)	176 (25.1%)	438 (62.5%)	0.034
WVU DFMB (n = 129)	4 (3.1%)	4 (3.1%)	25 (19.4%)	96 (74.4%)	

NICU rates were lower in the DFMB/ACE group (44.9%) compared to the non-DFMB/ACE group (49.2%) but this difference was not statistically significant. For birth modality, participants in the DFMB/ACE group had a lower cesarean delivery rate (34.6%) and a higher chance of vaginal birth (65.4%) compared to the non-DFMB/ACE group (cesarean 41.2%) and vaginal birth (59.8%). This difference was not statistically significant. Maternal hepatitis C was significantly higher in the DFMB/ACE group compared to the non-DFMB/ACE group (OR = 2.74 95 %CI 1.80 – 4.16).

## 4 Discussion

The findings of this study show that for women who used licit or illicit substances during pregnancy, their rates of low birth weight, premature delivery, and extreme premature delivery were lower for women enrolled in the DFMB/ACE program. Though a causal relationship cannot be established due to the retrospective observational study design, the findings suggest that the DFMB/ACE program may have potentially improved birth outcomes for mothers and their infants. Prematurity leads to long-term complications for children and, as such, any reduction in its incidence should not be taken lightly (Pravia, 2020). Given causes of premature delivery are multifactorial it is hard to pinpoint one aspect of the DFMB program that likely contributed predominantly to this outcome. We believe there is a multifaceted explanation that surrounds the DFMB patients’ decreased use of illicit substances, increased medical access, and improved social support.

The DFMB/ACE program is tailored to provide individualized care for pregnant women. There are various programs in the area that serve to provide MOUD to patients. Of our non-DFMB/ACE cohort, 257 patients were involved in treatment programs for substance use disorder with 98.1% using MOUD during their pregnancy. This group had birth outcomes essentially identical to the overall cohort. This leads us to believe that involvement in the DFMB group was a significant contributor to improved birth outcomes.

Both groups had similar rates of NAS. For women who are treated with MOUD, NAS can be anticipated and adequately treated. Initial treatment of NAS involves nonpharmacologic interventions with breastfeeding contributing to decreased severity of NAS. (Anbalagan, 2022) Women in the DFMB/ACE group had a higher incidence of breastfeeding at discharge, which may be due to increased education and support during the prenatal period. Though NAS has been correlated to poor long-term outcomes for infants, it is hard to attribute those outcomes specifically to maternal opioid use as various factors contribute to these infant outcomes including prematurity, low birth weight, polysubstance use, and low socioeconomic status. (Anbalagan, 2022) Studies have shown that polysubstance use contributes to more severe NAS scoring than buprenorphine alone, which is why close follow-up and MOUD are standards of care for pregnant women with substance use disorder (Nida, 2019).

Those in the DFMB/ACE group also had a 6.6% lower cesarean delivery rate compared to the non-DFMB/ACE group. While this difference was not statistically significant, it is possible that the DFMB/ACE group was underpowered to detect a difference. Further study of the ongoing program would be needed to evaluate this finding in the future.

Our study also demonstrated the higher prevalence of polysubstance within the non-DFMB/ACE group. Participants within the DFMB/ACE group have access to peer recovery support specialists that can aid in their reduction in the use of substances both licit and illicit. Tobacco was a common substance utilized by women in our study. Tobacco is highly addictive, and it is commonly used with other illicit drugs including cannabis. (Anderson and Chan, 2016, Sharp and Chen, 2019). The women in the DFMB/ACE group had a lower incidence of cannabis and tobacco use during pregnancy compared to the non-DFMB/ACE cohort. Tobacco smoking cessation is one of the key elements that is reinforced as part of the DFMB/ACE group. Continuous monitoring and referring women for tobacco cessation are interventions on which this program focusses. These types of interventions have shown to be effective; a Cochrane review including 30 articles concluded that there is high-quality data that counseling in the late pregnancy can reduce smoking (RR 1.44 with CI 1/19 to 1.73). Counseling is an essential tool in smoking cessation that can improve perinatal outcomes, as the same review demonstrated a 17% reduction in low birth weight (Chamberlain, 2017).

We observed a higher incidence of polypharmacy even in the non-intervention group who were enrolled in outside substance use treatment programs. As polysubstance use is common within our study, it is difficult to tease out the specific impacts of each particular substance on neonatal outcomes. However, some researchers have discussed that there could be potential additive effects of pharmacologic (multiple drugs), lifestyle, or socioeconomic factors on perinatal outcomes. (Oga et al., 2018) Even after adjusting for socio-demographic variables, our study demonstrated a significant association between birthweight and gestational age outcomes between the two groups.

Another interesting finding in our study was the higher proportion of patients with private insurance in the non-DFMB/ACE group (28.2%) compared to the DFMB/ACE group (3%). Out-of-pocket expenses for treatment for substance use disorder can be expensive and as such, we would expect patients with private insurance to have more access to MOUD or other substance use disorder therapies during pregnancy. But this was not observed in our study. (Patrick et al., 2020) It is interesting to note that despite many women undergoing supervised MOUD therapy during pregnancy, other support systems were not included or discussed compared to the women who were part of the DFMB/ACE program. As part of our findings in this study, we are contemplating investigating the differences between the interventions offered in the DMFB/ACE program and conventional MOUD programs. Over 10% of patients with private insurance reported illicit opioid use during pregnancy. This shows that even patients who are financially more likely to afford care could still benefit from a program like DFMB. This study also found that nearly 74% of the women enrolled in the DFMB/ACE program were unemployed which is consistent with the higher rate of Medicaid coverage. It is possible that employment could be a barrier to women participating in the DFMB/ACE program as it requires consistent meetings with support groups, psychiatrists, and peer recovery support specialists. Further studies are required to confirm these findings and investigate possible variables that may affect employment status including substance use and poor prenatal care.

Our study did not examine specific reasons for why some women elected and some refused to participate in the program. Low income could be a stressor that can affect birth weight among other prenatal outcomes (Borders et al., 2007). The DFMB/ACE participants were predominantly from a lower socioeconomic class, yet they had higher birth weights and reached later gestational ages than the non-intervention group. This suggests that the support and treatment provided by the program contributed to improved outcomes. We believe the women who had less social and financial support before being offered involvement in the program were more likely to accept. This theory is multi-layered and would require additional research to evaluate.

Considering the higher prevalence of hepatitis C in women with a history of substance abuse, we explored this relationship in our study. We noticed that hepatitis C prevalence was higher in the DFMB/ACE group compared to the non-DFMB/ACE group. One probable explanation for this is increased screening for Hepatitis C among the participants because of the comprehensive care provided as part of the DFMB/ACE program. DFMB/ACE participants were more likely to be identified as having a substance use disorder early in their pregnancy and provided additional services as part of the program.

### 4.1 Weakness and strengths of the study

Due to the retrospective study design, there is no data available on how many women from the non DFMB/ACE group were identified during pregnancy and offered the program. Additionally, there is no data on how many women who were offered the program declined and if they declined, their reasons for not participating in the DFMB/ACE project. The demographics, while likely representative of the Appalachian region, are not diverse and thus not generalizable to other geographical areas. A future project to determine if all women with substance use are identified during prenatal visits and offered the program, an expoloration of why women decline participation in DFMB/ACE, as well as the perinatal outcomes in these groups would be of value. Due to database changes, a proportion of DFMB/ACE participant data was not available. This lessened the intervention group evaluation. Additionally, the research team did not have long-term data from newborns after discharge from the hospital for the non-intervention group.

The study has several strengths. First, women in both groups were within the same geographical area and delivered at the same hospital. We were able to examine the variations of several outcomes between the two groups and understand the sociodemographic differences and similarities between them. While the data in the intervention group was limited to 134 participants, we were able to demonstrate the potential positive impact of the DFMB/ACE program.

## 5 Conclusions and future implications

Women in the DFMB/ACE group appear to have higher birth weights than women who were not participants in the DFMB/ACE program. Long-term data from the infants correlating with the DFMB/ACE intervention is necessary to understand the possible future implications and expand this intervention to other women across the Appalachia region. It would also be worthwhile to investigate whether women using licit and illicit substance are identified during pregnancy as well as the reasons that women elected not to participate in the DFMB program to provide adjustments to bolster participation and ensure a correlation between the program and the improvement of perinatal outcomes.

We also recognize that not all women experienced this program in the same way. They were brought in at different stages of pregnancy and recovery. It would be interesting in future studies to look at how different experiences within the program contributed to different neonatal outcomes. Ideally, patients who could benefit from this intervention would be recognized before becoming pregnant to reduce potential adverse outcomes. A major focus of the program is to expand outreach efforts to improve the number of prenatal visits and engage mothers in their pregnancies, recognizing that polysubstance use may impact their babies and their lives. In addition to the comprehensive needs assessment and coordination of care that DFMB/ACE provides to participants, another strength of the program are the services provided by the peer recovery support specialist. The assistance provided by a person with lived experience helps reduce stigma and provides an extra layer of support that many pregnant women with substance use disorders lack.

## Funding

This study was funded by the West Virginia Perinatal Partnership.

Dr. Dueñas-Garcia Omar is a consultant for Applied medical- no financial disclosures from that company are relevant to this manuscript.

## Declaration of Competing Interest

The authors declare the following financial interests/personal relationships which may be considered as potential competing interests: Dr. Dueñas-Garcia is a consultant for Applied Medical. The rest of the authors have nothing to disclose.

## Data Availability

Data will be made available on request.
