# Supplementary material for: Perinatal outcomes in women referred to the West Virginia University Assist Connect and Encourage (ACE) – A program of the Drug Free Moms and Babies Project (DFMB) for women with substance use during pregnancy
Source: Prev Med Rep. 2023 Jul 4;35:102312. doi: 10.1016/j.pmedr.2023.102312 (PMC10339237; doi:10.1016/j.pmedr.2023.102312)
Supplement: Supplementary data 1 [file mmc1.pdf]

WEST VIRGINIA Department of Health & Human Resources BUREAU FOR PUBLIC HEALTH Office of Maternal, Child and Family Health

WEST VIRGINIA
PRENATAL RISK SCREENING INSTRUMENT

Last Name:	First Name:	MI:	Date of Birth: - -	Social Security #: - -

Street:	City:	State:	Zip Code:	County of Residence:	Telephone:	Maiden Name:

Race: **(Check all that apply)**
☐ **White** ☐ **Black/African American** ☐ **Asian**
☐ **American Indian/Alaska Native**
☐ **Native Hawaiian/Pacific Islander**

Ethnicity: ☐ Hispanic/Latino ☐ Not Hispanic/Latino

U.S. Citizen: ☐ **Yes** ☐ **No**

Married: ☐ **Yes** ☐ **No**

Insurance Source:
☐ **Health Insurance:** ____________
☐ **No Insurance**
☐ **Medicaid #:** ____________

Date of 1st Prenatal Visit: (MM/DD/YYYY) ____ - ____ - ________

Current Weight (lbs) : ☐☐☐

Height (Ft-inches) : ☐ – ☐☐

Blood Pressure: ☐☐☐ / ☐☐☐

OBSTETRICAL HISTORY:

Gravida	Para				
	Term	Pre	SAB	EAB	Live

LMP: (MM/DD/YYYY): ____ - ____ - ________

EDC: (MM/DD/YYYY): ____ - ____ - ________

Date of Last Delivery: ____ - ____ - ________

Type of Delivery: 1st Trimester: ☐ Miscarriage ☐ Abortion
2nd Trimester: ☐ Miscarriage ☐ Abortion
☐ Preterm Birth ☐ Term Birth

ORAL HEALTH:

Sensitive/Bleeding Gums	☐ Yes	☐ No
Loose/Broken/Decayed Teeth	☐ Yes	☐ No
Dental visit within the last year	☐ Yes	☐ No
Dental cleaning in the last year	☐ Yes	☐ No

BREASTFEEDING

Do you intend to breastfeed?	☐ Yes	☐ No
Are you currently breastfeeding?	☐ Yes	☐ No

PREGNANCY RISK FACTORS	Current Preg. Y	Current Preg. N	Prior Preg. Y	Prior Preg. N
PROM	na	na	☐	☐
Preterm Delivery	na	na	☐	☐
Cervical Incompetence	☐	☐	☐	☐
Cervical Surgery	☐	☐	☐	☐
Macrosomia >4000 grams	☐	☐	☐	☐
Gestational Diabetes	☐	☐	☐	☐
Previous Stillbirth	na	na	☐	☐
Oligohydramnios	☐	☐	☐	☐
Opioid Abuse Treatment	☐	☐	☐	☐
Hypertension	☐	☐	☐	☐
PIH/Preeclampsia	☐	☐	☐	☐
IUGR	☐	☐	☐	☐
Low Birth Weight <2500 grams	☐	☐	☐	☐
Ectopic Pregnancy	☐	☐	☐	☐
Placental Previa	☐	☐	☐	☐
Placental Abruption	☐	☐	☐	☐
Previous C-Section	na	na	☐	☐
Hepatitis B	☐	☐	☐	☐
Hepatitis C	☐	☐	☐	☐
Pyelonephritis	☐	☐	☐	☐
Chromosome Abnormalities	☐	☐	☐	☐
Fetal Anomaly	☐	☐	☐	☐
Short Cervix	☐	☐	☐	☐

Other/Unlisted Risk Factor: ____________

Bleeding during current pregnancy?
Trimester: ☐ 1st ☐ 2nd ☐ 3rd ☐ No

FAMILY HISTORY:	Current Preg. Y	Current Preg. N	Prior Preg. Y	Prior Preg. N	Family Hist. Y	Family Hist. N
Multiple Gestation	☐	☐	☐	☐	☐	☐
Fetal Genetic/Structural Abnormalities	☐	☐	☐	☐	☐	☐

MEDICAL CONDITIONS:	Yes	No	On Meds
High Blood Pressure	☐	☐	☐
Kidney Disease	☐	☐	☐
Diabetes	☐	☐	☐
Asthma	☐	☐	☐
Heart Condition	☐	☐	☐
Thyroid Disease	☐	☐	☐
Clotting Disorder	☐	☐	☐
STD	☐	☐	☐
Seizures	☐	☐	☐
Rh Negative	☐	☐	☐
Other: ____________	☐	☐	☐

PSYCHOSOCIAL RISK FACTORS:	Yes	No
Disabled	☐	☐
Unemployed/Inadequate Income	☐	☐
Husband/Partner Employed	☐	☐
Homeless	☐	☐
Unstable Housing	☐	☐
Education <12 years	☐	☐
Currently in Foster Care	☐	☐
Inadequate Transportation	☐	☐
Inadequate Social Support	☐	☐
Unplanned Pregnancy	☐	☐
Do you have enough to eat	☐	☐
Eating Disorder	☐	☐
Difficulty with Reading and Understanding	☐	☐
Internet Access	☐	☐

ENVIRONMENTAL RISK FACTORS:	Yes	No
Lead: House Built before 1978	☐	☐
Viral: Cats or Birds in Home	☐	☐
Tobacco: 2nd or 3rd Hand Smoke	☐	☐

REASONS FOR LATE ENTRY INTO PRENATAL CARE: (check all that apply)

Does not apply	☐	Financial	☐
Insurance Enrollment Delay	☐	Child Care Issues	☐
Unaware of Importance of PNC	☐	Access to pregnancy testing	☐
Couldn't find a health provider	☐	Transportation	☐
Abortion desired/unsuccessful	☐	Other: ____________	

Have either of your parents had a problem with **drugs?** Yes ☐ No ☐ **or alcohol?** Yes ☐ No ☐
Has your partner had a problem with **drugs?** Yes ☐ No ☐ **or alcohol?** Yes ☐ No ☐
In the past, have you had a problem with **drugs?** Yes ☐ No ☐ **or alcohol?** Yes ☐ No ☐
During this pregnancy, have you used **drugs?** Yes ☐ No ☐ **or alcohol?** Yes ☐ No ☐

Have you ever smoked cigarettes? Yes ☐ No ☐
Do you currently smoke cigarettes? Yes ☐ No ☐
If yes, # of cigarettes per day: ________
I quit (when): ________
Does your partner smoke? Yes ☐ No ☐

Have you ever been a victim of abuse or violence? Yes ☐ No ☐
Has your partner's anger ever worried or scared you? Yes ☐ No ☐
Have you ever felt down or hopeless? Yes ☐ No ☐
Have you lost interest in things you used to do for fun? Yes ☐ No ☐

In the month before you knew you were pregnant, did you take prescription drugs? Yes ☐ No ☐
If YES, what were the drugs? ____________
Who prescribed the drugs?

Provider Name and Title: (print)	Provider Signature:	Person Completing the form:	Provider Telephone No.:	Date:

I am interested in further follow-up. I give my consent for necessary referrals to be made. I understand that my participation in any referral services is voluntary and that all information provided will be held strictly confidential.

Patient Name: (print)	Patient Signature:	Date: - -

7317074

WVDHHR/BPH/OMCFH/DPWH PRSI 12/29/2016

PLEASE COMPLETE AND FAX TO (304) 957-0176

WV PRENATAL RISK SCREENING INSTRUMENT INSTRUCTIONS

The Prenatal Risk Screening Instrument (PRSI) is intended to promote early and accurate identification of prenatal risk factors. Prenatal Risk Screening Instrument is to be completed by the physician/clinician at the first prenatal visit. If the patient answers "Yes" to any pregnancy or medical risk factor, a Maternal Fetal Medicine consultation should be considered. Data gathered through the PRSI will be used to develop procedures, policy, and obtain funding to address prenatal risk. The goal is to improve birth outcomes for mother and infant. Completion and submission of this form is required by State Law.

General Instructions
Print clearly. Complete the form accurately and completely. When asked to select "Yes" or "No", choose only one option.
Patient Information
Name (List patient's Last Name, First Name & Middle Initial)
Date of Birth (List patient's date of birth as MM/DD/YYYY)
Social Security Number (List patient's social security number; if patient is undocumented or a non-citizen use 000-00-0000)
Address (Use current address where the patient resides)
County of Residence (List the West Virginia County that patient's address is located)
Telephone Number (Use a current telephone number & alternate number, if applicable, where patient can be reached)
Maiden Name (her last name given at birth)
Race/Ethnicity (Check all that apply)
U.S. Citizen (Choose only one option)
Married (Choose only one option)
Insurance Source (Select type of insurance source that patient currently has; if Medicaid, list Medicaid number; private insurance, list insurance company name, ex: PEIA, BCBS)
Entry into Prenatal Care
Date of First Prenatal Visit (Enter the date of the patient's initial medical examination during this pregnancy)
Current Weight (List patient's current weight in pounds)
Height (List patient's current height in feet/inches)
Blood Pressure (List patient's blood pressure reading at time of this visit)
Obstetrical History
Gravida (Enter # of pregnancies in the boxes; include current pregnancy in this number. If Gravida >1, the Para field must be completed.)
Para (This is the # of: Term=Term Deliveries; Pre=Preterm Deliveries; SAB=Spontaneous Abortions; EAB=Elective Terminations; & L=Live Births)
LMP (List date of last menstrual period)
EDC (List estimated date of confinement)
Date of Last Delivery (List patient's last pregnancy delivery date, if applicable)
Type of Delivery (Select type of delivery patient had from last pregnancy, if applicable)
Oral Health
Select "Yes" or "No". If patient answers "Yes" to any of the questions, please consider a referral to a dentist or provide patient education.
Breastfeeding
Select "Yes" or "No" to the questions regarding breastfeeding.
Pregnancy Risk Factors
Select "Yes" or "No" to indicate the presence of risk factors in the patient's current and/or prior pregnancy(ies).
Bleeding During Current Pregnancy
If "Yes", select the trimester(s) that bleeding occurred. Select "No" if bleeding did not occur.
Family History
Select "Yes" or "No" to indicate the presence of risk factors in the patient's current and/or prior pregnancy(ies) and/or whether there is a family history for the selected risk factors.
Medical Conditions
Select "Yes" or "No" to indicate whether the patient currently has the listed medical risk factor and/or is taking medication for the condition.
Psychosocial Risk Factors
Select "Yes" or "No" for each risk factor listed.
Environmental Risk Factors
Indicate by selecting "Yes" or "No" whether the patient has been exposed to listed items in their environment. A patient who lives in a house built before 1978 is at risk for exposure to lead paint.
Reasons for Late Entry into Prenatal Care
Complete this section only when a patient enters prenatal care in the 2nd or 3rd trimester. Fill in "Yes" for all reasons that apply.
Additional Screening Questions
These questions are used as a screening tool to begin discussion about use of drugs, alcohol, tobacco and/or abuse. Advise the patient that the responses she provides are confidential and may only be used for her evaluation and treatment. Any patient who answers "Yes" to one or more questions may warrant further assessment and follow-up.
Provider Information
List name, title, and telephone number of provider completing the PRSI; list date the form was completed. **PLEASE PRINT CLEARLY**
Person Completing Form
List name; Please Print Clearly
Consent
Patient's participation in any referral services is voluntary and her consent must be provided. If patient is interested in further followup/referrals, she must print name, sign and date the form. If patient is not interested in referral services, please leave this section blank.

Completion

Fax the form to (304) 957-0176. Do not include coversheets. Check to be sure the correct side of the form is transmitted. Fax only one form per patient; do not re-fax a patient's form. Duplicate faxes create problems with processing.

If a payer (MCO, Insurance, etc.) requests the PRSI be faxed to their office, the State Law requires submission to the Office of Maternal, Child and Family Health.

If you have questions, concerns or would like technical assistance to complete the PRSI, please call 304-356-4397.
